# High-content screening identifies a role for Na^+^ channels in insulin production

**DOI:** 10.1098/rsos.150306

**Published:** 2015-12-02

**Authors:** Marta Szabat, Honey Modi, Reshma Ramracheya, Vroni Girbinger, Forson Chan, Jason T. C. Lee, Micah Piske, Sepehr Kamal, Yu Hsuan Carol Yang, Andrea Welling, Patrik Rorsman, James D. Johnson

**Affiliations:** 1 Department of Cellular and Physiological Sciences, University of British Columbia, Life Sciences Centre, 2350 Health Sciences Mall, Vancouver, British Columbia, Canada V6T 1Z3; 2 Oxford Centre for Diabetes Endocrinology and Metabolism, University of Oxford, Churchill Hospital, Headington OX3 7LE, UK; 3 Institut für Pharmakologie und Toxikologie der Technischen Universität, 80802 München, Germany

**Keywords:** islet beta-cells, insulin synthesis, live-cell imaging, sodium channels, high-content screening

## Abstract

Insulin production is the central feature of functionally mature and differentiated pancreatic *β*-cells. Reduced insulin transcription and dedifferentiation have been implicated in type 2 diabetes, making drugs that could reverse these processes potentially useful. We have previously established ratiometric live-cell imaging tools to identify factors that increase insulin promoter activity and promote *β*-cell differentiation. Here, we present a single vector imaging tool with eGFP and mRFP, driven by the *Pdx1* and *Ins1* promoters, respectively, targeted to the nucleus to enhance identification of individual cells in a high-throughput manner. Using this new approach, we screened 1120 off-patent drugs for factors that regulate *Ins1* and *Pdx1* promoter activity in MIN6 *β*-cells. We identified a number of compounds that positively modulate *Ins1* promoter activity, including several drugs known to modulate ion channels. Carbamazepine was selected for extended follow-up, as our previous screen also identified this use-dependent sodium channel inhibitor as a positive modulator of *β*-cell survival. Indeed, carbamazepine increased *Ins1* and *Ins2* mRNA in primary mouse islets at lower doses than were required to protect *β*-cells. We validated the role of sodium channels in insulin production by examining Nav1.7 (*Scn9a*) knockout mice and remarkably islets from these animals had dramatically elevated insulin content relative to wild-type controls. Collectively, our experiments provide a starting point for additional studies aimed to identify drugs and molecular pathways that control insulin production and *β*-cell differentiation status. In particular, our unbiased screen identified a novel role for a *β*-cell sodium channel gene in insulin production.

## 1. Introduction

Insulin production and secretion from pancreatic *β*-cells is carefully regulated to sustain normoglycaemia. However, type 1 or type 2 diabetes develops when circulating insulin levels drop below the threshold that marks the body's minimum requirements for glucose homeostasis. In type 1 diabetes, this occurs when *β*-cells are almost entirely destroyed via autoimmune attack [1]. In type 2 diabetes, *β*-cell exhaustion is thought to occur in the context of high-demand coincident with systemic insulin resistance [1,2]. We have also shown that insulin production, via effects on basal circulating insulin, is a key determinant of diet-induced obesity [3]. Thus, changes in insulin production have critical pathophysiological consequences.

A defining feature of a fully differentiated *β*-cell is robust insulin production. This truism has guided efforts to produce *β*-cell replacements from human embryonic stem cells [4]. Several groups, including ours, have recently explored the concept of *β*-cell dedifferentiation [5–14], and elegant *in vivo* work has established *β*-cell dedifferentiation as a new mechanism for *β*-cell failure in uncontrolled type 2 diabetes [15–17]. We have shown previously that the differentiation state can be measured in real-time at the single-cell level using a fluorescent reporter of *Ins1* promoter activity in rodent and human *β*-cells [5,7,9,18]. Specifically, our strategy employed a single ‘ratiometric’ dual-reporter lentivirus wherein eGFP was driven by a fragment of the *Ins1* promoter and mRFP was driven by a fragment of the *Pdx1* promoter [7]. However, this original dual-reporter construct employed untargeted fluorescent proteins that distributed across the whole cell cytoplasm and nucleus, limiting its use for applications requiring automated cell counting. Thus, in this study, we sought to generate an improved imaging tool for assessing insulin production and differentiation state in living *β*-cells.

Here, we report the development of an improved *Ins1*/*Pdx1* dual-reporter lentivirus and validate it against our previously published tool. We employ this new reporter construct in a multi-parameter, high-content screen of off-patent drugs and identify several small molecules capable of regulating *β*-cell maturity and/or insulin gene expression, without significant effects on cell viability. Notably, we identified carbamazepine, an ion channel-modulating drug we have previously shown to have favourable effects on *β*-cell survival, as a small molecule capable of driving *Ins1* promoter activity in MIN6 cells and increasing insulin mRNA expression in primary islets. Collectively, our observations form the basis for additional studies on numerous factors, including carbamazepine.

## 2. Material and methods

### 2.1 Construct design and cloning

The original pTiger*Pdx1*mRFP/*Ins1*eGFP construct was cloned as previously described [7]. To generate the new nuclear-targeted pTigerPdx1mRFP-NLS/Ins1eGFP-NLS vector, the nuclear localization signal (NLS) from simian virus large T-antigen (sequence: ’GATCCAAAAAAGAAGAAAGGTA) was synthesized in tandem using custom oligos (Integrated DNA Technologies, IA, USA) flanked by compatible ends for downstream ligations. The single-stranded oligos were annealed and incorporated at the 3^′^ ends of each of the mRFP and eGFP sequences preceding the stop codons in the original vector.

### 2.2 Cell culture, primary islet isolation and fluorescence-activated cell sorting

Prior to experiments, cultures of the insulin-secreting MIN6 pancreatic cell line (used between passages 20 and 30) were grown in Dulbecco's modified eagle's medium (DMEM) containing 25 mM glucose, 100 units ml^−1^ penicillin, 100 μg ml^−1^ streptomycin and 10% vol/vol FBS (Invitrogen, Thermo-Fisher, Waltham, MA, USA). Primary mouse pancreatic islets were isolated from approximately 12 week-old male C57BL/6J mice (Jax, Bar Harbor, MA, USA) using the collagenase and filtration technique described previously [19,20]. Mice were housed in accordance with University of British Columbia Animal Care Committee guidelines. Islets were hand picked and cultured overnight (37°C, 5% CO_2_) in RPMI1640 medium (Invitrogen) with 11.1 mM glucose, 100 units ml^−1^ penicillin, 100 μg ml^−1^ streptomycin (Invitrogen) and 10% vol/vol FBS (Invitrogen), before being treated with various factors.

Fluorescence-activated cell sorting (FACS) was conducted on an Influx sorter (Cytopeia) at the Life Sciences Institute core facility. Cells were excited with a 488 nm laser (530/40 emission) and a 561 nm laser (610/20 emission).

### 2.3 Live-cell imaging and high-content screening

Standard live-cell imaging was performed in phenol red-free DMEM (25 mM) supplemented with 10% vol/vol FBS (Invitrogen) using a Zeiss 200M microscope as described previously [19]. For high-throughput imaging, MIN6 cells stably expressing the lentiviral constructs were seeded into 96-well plates and cultured as described [20,21]. MIN6 cells stably expressing the nuclear-targeted dual reporter were used for high-content screening. Cells were counter-stained with 50 ng ml^−1^ Hoechst 33342 (Invitrogen). Baseline culture conditions were phenol red-free DMEM (Invitrogen) containing 5 mM glucose (Sigma), 0.2% (wt/vol) BSA (Stem Cell Technologies, Vancouver, Canada), 100 units ml^−1^ penicillin, 100 μg ml^−1^ streptomycin (Invitrogen) and 4 mM glutamine (Invitrogen), unless otherwise indicated. Some control wells contained media with 25 mM glucose. Other control wells contained media with activin A (4 nM) or follistatin (100 nM; R&D Systems, Minneapolis, MN, USA), which we have previously shown can modulate the ratio of *Ins1* promoter activity to *Pdx1* promoter activity [5]. In some control wells, media was supplemented with 10% vol/vol FBS. Other control wells included a cytokine cocktail consisting of 25 ng ml^−1^ TNF-α, 10 ng ml^−1^ IL-1*β*, 10 ng ml^−1^ IFN-γ (R&D Systems). Following 48 h treatments, cells were imaged with ImageXpress^MICRO^ (Molecular Devices, Sunnyvale, CA, USA) at 37°C and 5% CO_2_. Image analysis was carried out using MetaXpress Software (Molecular Devices) [22,23]. The ‘self-organizing maps’ function of AcuityXpress (Molecular Devices) was employed to identify compounds with similar activities [23].

The Prestwick Library of 1120 drugs includes a diverse array of chemicals, including off-patent drugs (www.prestwickchemical.com). Compounds were pinned from 96-well round bottom plates (Corning) at approximately 15 μM using a 0.7 mm diameter 96-pin tool equipped onto a Biorobotics Biogrid II robot. The final compound concentration was approximately 8.5 μM, and we recognize that this would not have been an ideal dose for all drugs in the library.

### 2.4 Quantitative PCR and insulin content measurements

Following 24 h of drug treatment, total RNA was isolated from mouse primary islets using RNeasy mini kit (Qiagen, Mississauga, ON, Canada). cDNA was generated using qScript cDNA SuperMix (Quanta Biosciences, Gaithersburg, MD, USA) and quantitative RT-PCR was conducted using *Ins1* (FW—5^′^-TCAGAGACCATCAGCAAGCA-3^′^, RV—5^′^-GGGACCACAAAGATGCTGTT-3^′^), *Ins2* (FW - 5^′^-GGAGCGTGGCTTCTTCTACA-3^′^, RV—5^′^-CAGTGCCAAGGTCTGAAGGT-3^′^), *Pdx1* (FW—5^′^-GACCTTTCCCGAATGGAACC-3^′^, RV—5^′^-GTTCCGCTGTGTAAGCACC-3^′^), *β*-*actin* (FW—5^′^-TGCGTGACATCAAAGAGAAG-3^′^, RV—5^′^-GATGCCACAGGATTCCATA-3^′^) primers (Integrated DNA Technologies, Coralville, IA, USA) and PerfeCTa qPCR SuperMix (Quanta) on a StepOnePlus instrument (Applied Biosystems). Relative gene expression changes were analysed using the 2 to the negative delta-delta Ct method, with *β*-actin as the reference gene.

Insulin content was measured after acid ethanol extraction in the same number of islets of similar size from approximately 1 year old female Nav1.7 (Scn9a) knockout mice, which have previously been characterized [24].

### 2.5 Electrophysiology

HEK 293 cells were cultured in Quantum 286 complete medium with l-glutamine and penicillin/ streptomycin. One day before transfection, they were seeded on polylysine-coated glass coverslips. The sodium channel Nav1.7 (NM_002977.3) was transiently transfected with a pcDNA3 expression vector (total cDNA 0.225 μg/coverslip) by using Lipofectamine^TM^ 2000 according to the manufacturer's guide (Life Technologies, Thermo-Fisher). After transfection, the cells were grown at 37°C, 5% CO_2_ for 40–48 h before beginning electrophysiological experiments. Islets were isolated according to previously published protocols [25].

Currents were recorded at room temperature from HEK 293 or single islet cells using the standard whole-cell technique. The measurements were performed using an EPC-9 patch-clamp amplifier (HEKA Electronics, Lambrecht/Pflaz, Germany) and Pulse (v. 8.54) software. Currents were compensated for capacitive transients with the built-in compensation. No leak compensation was done. Patch pipettes were pulled from borosilicate glass with a resistance of 2.8–4.0 MΩ when filled with the pipette solution. The pipette solution contained (mM): 120 CsCl, 2 MgCl_2_, 10 EGTA, 10 TEA-C, 10 HEPES, pH 7.2. The standard extracellular medium consisted of (mM): 150 NaCl, 2 KCl, 2 CaCl_2_, 1 MgCl_2_, 10 HEPES, 10 glucose, pH 7.4. Carbamazepine was dissolved as stock solution in DMSO and diluted to the final concentration in extracellular solution on each experimental day. Currents were measured by standard 100 ms depolarizations from a holding potential of −80 mV (HEK 293 cells) or −120 mV (islet cells). The steady-state inactivation properties of sodium currents were studied using a conventional two-pulse protocol in which a 30 ms test depolarization to −10 or 0 mV was preceded by a 100 ms conditioning pulse (*V*_pre_) to voltages between −140 and 0 mV (HEK 293 cells) or between −180 and −40 mV (islet cells). Data for steady-state inactivation were fitted by a Boltzmann equation:

$$\frac{I}{I_{\max}}=\frac{1-A}{1+\exp((V_{\mathrm{pre}}-V_{0.5})/k)}+A,$$

where *I*/*I*_max_ is the relationship between the current amplitudes (*I*) and the maximal current amplitude at the most negative *V*_pre_ (*I*_max_), *A* is the saturated level of *I*/*I*_max_, *V*_0.5_ is the potential at which *I*/*I*_max_ is half-maximal and *k* represents the slope factor. Dose–inhibition curves were calculated by a Hill equation. Electrophysiological data were analysed with Origin v. 6.1 (OriginalLab Corp., Northampton, MA, USA).

### 2.6 Statistical analysis

Data of replicated experiments are expressed as mean±s.e.m., unless otherwise indicated. Results were considered statistically significant when *p*<0.05 using Student's *t*-test.

## 3. Results

### 3.1 Development of a nuclear-targeted *Ins1*/*Pdx1* dual-reporter lentivirus

**Figure 1.**
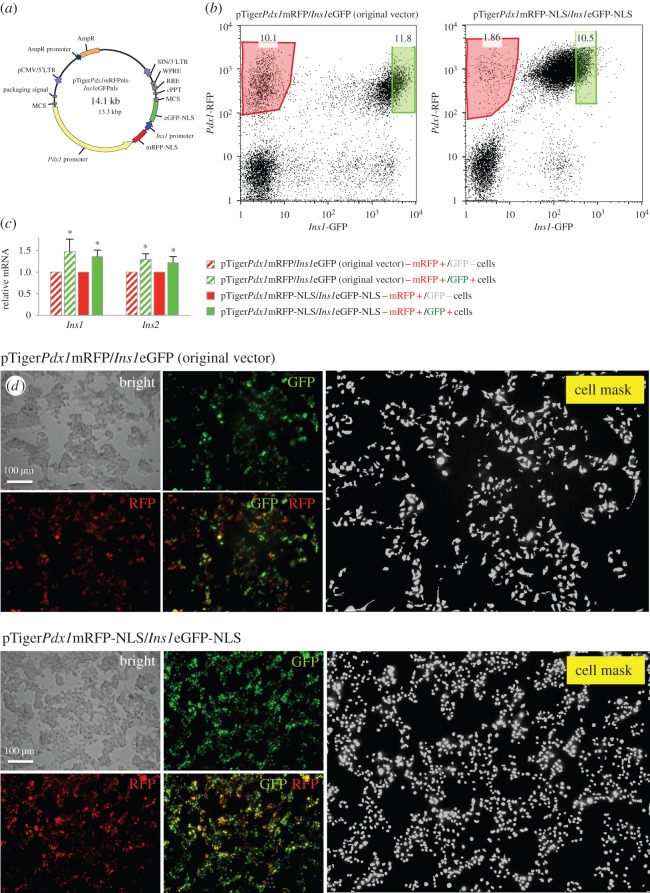
Targeting fluorescent reporters for *Pdx1* and *Ins1* to the nucleus and validation. (*a*) Nuclear localization sequences were added to mRFP and eGFP fluorescent proteins for nuclear targeting. (*b*) FACS plots of MIN6 cells infected with the original *Pdx1*/*Ins1* dual-reporter vector expressing cytoplasmic fluorescent proteins (left panel), and the new *Pdx1*/*Ins1* dual-reporter vector expressing nuclear-targeted fluorescent proteins (right panel). (*c*) *Insulin1* and *insulin2* mRNA were measured by qRT-PCR in vector-infected, FACS-purified *Pdx1*^+^/*Ins1*^−^ cells and *Pdx1*^+^/*Ins1*^+^ MIN6 cells (*n*=3), *p<0.05. *Pdx1* mRNA was not different between these cell populations. (*d*) Original *Pdx1*/*Ins1* dual-reporter vector labelled the cytoplasm of MIN6 cells (top panels), whereas the new nuclear-targeted reporters mark only the nucleus of labelled cells (bottom panels).

Fluorescent proteins driven by known promoter sequences provide windows into gene expression at the single-cell level [8]. The inclusion of green fluorescent and red fluorescent promoter reporters on the same lentiviral backbone in our first construct ensured that each cell had the potential to express both fluorescent proteins and permitted ratiometric analyses of signals with a known stoichiometry [5–7,9,18]. To improve upon our original dual reporter, especially for automated cell counting applications, we targeted eGFP and mRFP to the nucleus by way of a standard NLS (figure 1*a*). Consistent with the behaviour of the original dual-reporter lentivirus, FACS purification of cells infected with the nuclear-targeted dual-reporter displaying only *Pdx1* promoter activity (i.e. mRFP-positive without significant eGFP signal) had significantly less *Ins1* and *Ins2* mRNA when compared with cells expressing robust *Ins1* promoter activity (figure 1*b*,*c*). As expected, the nuclear-targeted dual-reporter construct was advantageous for delineating single cells, even in highly clumped cultures and at low magnification (figure 1*d*). Based on these studies, we have confidence that the nuclear-targeted *Ins1*/*Pdx1* dual promoter reporter behaves in a similar fashion to our original imaging tool, with the notable improvement of nuclear targeting.

### 3.2 High-content screening for drugs that alter *Ins1* and/or *Pdx1* promoter activity

We assessed the utility of this new tool by conducting a three-parameter high-content screen to identify drugs that might increase insulin expression. We chose MIN6 cells for this high-content screen because they express lower levels of insulin relative to primary cells in our hands. Others have employed subdifferentiated cell models in similar screens [26], as it is easier to see increases in *Ins1* promoter activity under such conditions. We chose the Prestwick library of off-patent drugs because mechanisms of action are known for many of the compounds [23]. Prior mechanistic knowledge has the potential to provide insights into the basic biology of insulin expression in *β*-cells.

**Figure 2.**
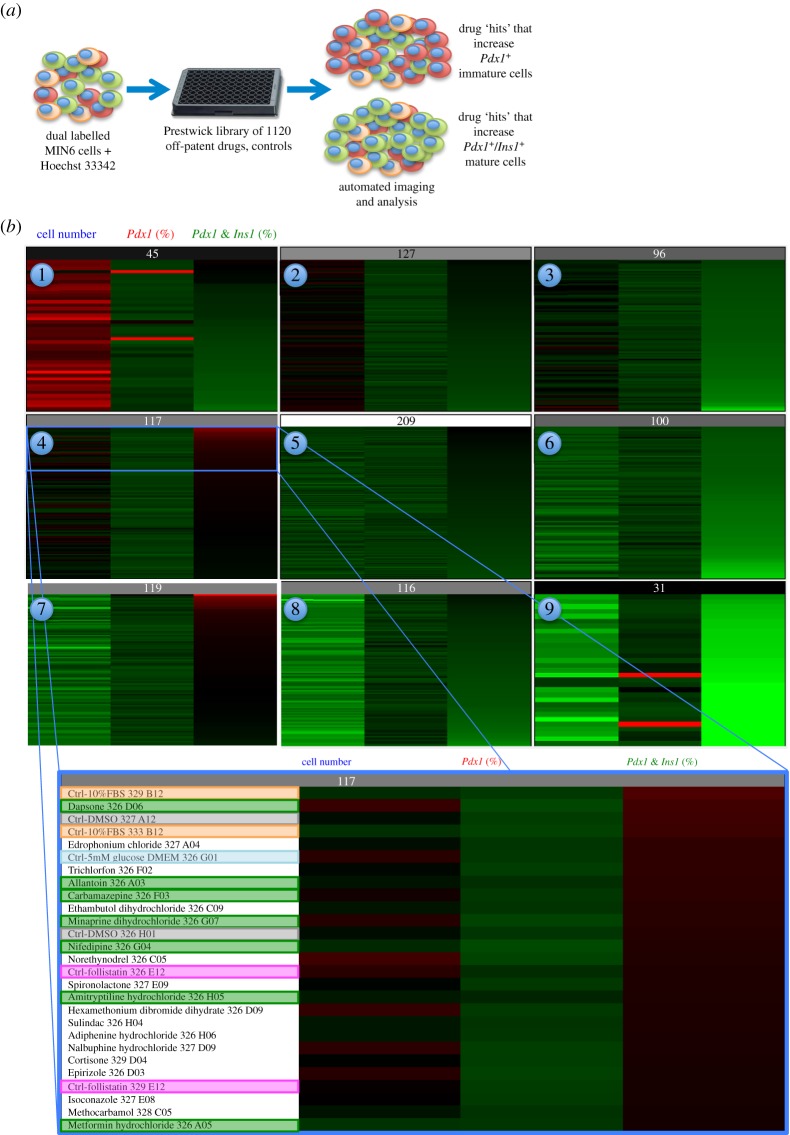
Self-organizing response groupings of a three-parameter high-content screen to assay *Ins1* promoter activity, *Pdx1* promoter activity and cell proliferation/survival. (*a*) Experimental design for a three-parameter, high-throughput imaging screen using MIN6 *β*-cells. (*b*) Self-organizing heat maps of nine result groups generated with AcuityXpress software. In these heat maps, red represents high and green represents low. Screen hits were chosen based on their clustering with positive controls. For example, follistatin is a positive control for a factor that increases *Ins1* promoter activity and *β*-cell differentiation. The blow-up of group 4 (bottom panel) contains the hits that were followed up in this study.

MIN6 cells stably expressing the nuclear-targeted *Ins1*/*Pdx1* dual promoter reporter were continuously cultured in a low concentration of Hoechst 33342 during drug treatment (figure 2*a*), which we have previously shown does not have significant effects on cell survival [27]. These cells were treated with 1120 drugs as well as an array of controls. These controls included: DMSO controls, because the drugs were solubilized in DMSO; untreated 5 mM glucose controls (basal media); untreated 25 mM glucose controls; 10% FBS; a toxic cytokine cocktail of TNF-α, IL-1*β* and IFN-γ (control for pathophysiological inducers of *β*-cell death); follistatin or activin A (controls for increased and decreased *Ins1*/*Pdx1* ratio, respectively) [5,6]. We used a combination of automated and manual approaches to select ‘hits’ for further testing.

AcuityXpress software generated self-organizing maps that took into account the overall properties of each treatment, including cell number, percentage of *Pdx1*-positive but *Ins1*-low cells (‘immature cells’ [7]), and percentage of *Pdx1*-positive and *Ins1*-high cells (‘mature cells’ [7]), and then clustered similar treatments together (figure 2*b*). This approach resulted in nine response groupings. For example, response group 1 contained treatments that increased cell number, but were mostly low or neutral with regards to *Ins1* or *Pdx1* promoter activity. Response groups 2, 3, 5, 6 and 8 contained drugs with largely neutral effects on the parameters tested. Response group 7 contained treatments that increased *Ins1* promoter activity, but that also had deleterious effects on cell viability. We focused on response group 4 which contained 117 treatments that nominally increased *Ins1* promoter activity independent of changes in cell number. We specifically focused on the top approximately one-third of response group 4 where changes in *Ins1* promoter activity were particularly robust (figure 2*b*, bottom panel). This approach identified dapsone, allantoin, minaprine dihydrochloride, nifedipine, amitryptiline hydrochloride, metformin, carbamazepine as drugs of interest that cluster with positive controls such as 10% FBS and follistatin, which we have previously shown can increase *Ins1* promoter activity in *β*-cells [5].

**Figure 3.**
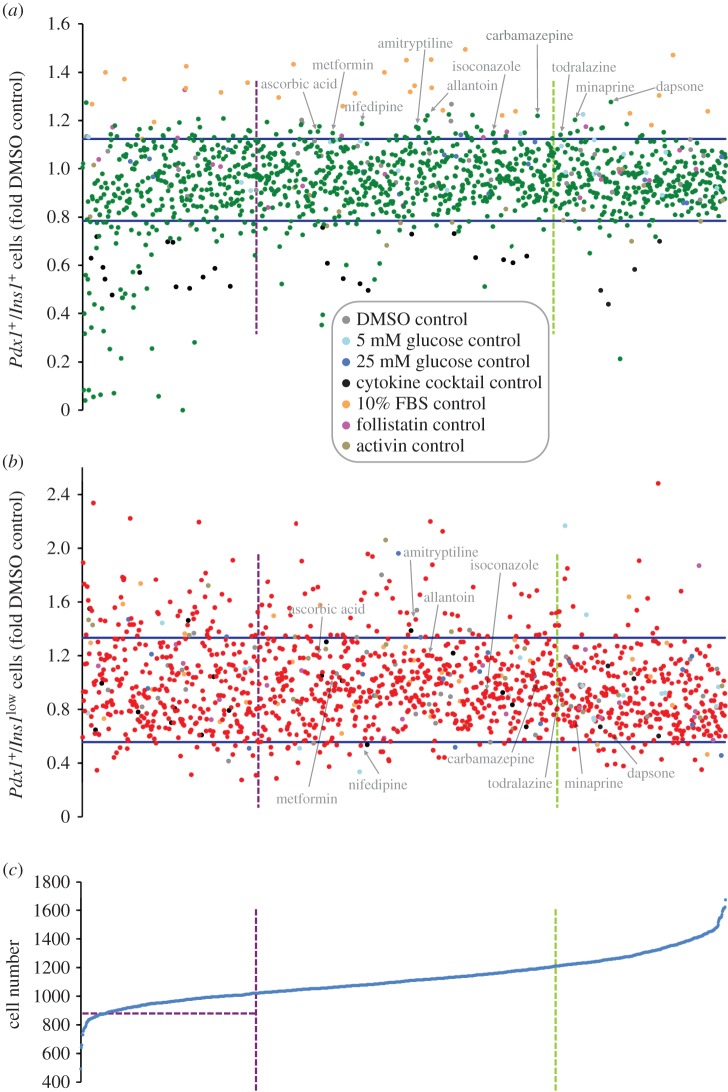
Effects of drugs on *Ins1* promoter activity, *Pdx1* promoter activity and cell proliferation/survival. (*a*–*c*) Data for the Prestwick library drugs for all three parameters is shown ranked according to their effects on increasing total cell number (bottom panel). Factors outside of the arbitrary purple and green vertical lines were deemed to have potential effects on cell viability and/proliferation. Drugs that fell outside of the blue lines in (*a*,*b*), which show 2xMAD (median absolute deviation), were then assessed as possible hits. Hits were chosen for their effects on several outputs such as inducing *β*-cell maturation by increasing the relative number of mature *Pdx1*^+^/*Ins1*^+^ cells (top) and concomitantly reducing the relative number of immature *Pdx1*^+^/*Ins1*^*low*^ cells (middle). Positive controls are marked in orange.

We next manually examined the *Ins1* promoter activity and *Pdx1* promoter activity data as a function of total cell number (figure 3). The data were normalized around the DMSO controls. We looked for treatments with *Ins1* promoter activity outside of two median absolute deviations (2MAD; figure 3*a*), but with relatively normal *Pdx1* promoter activity (figure 3*b*), indicative of a high *Ins1*/*Pdx1* promoter activity ratio. Dapsone, minaprine and todralazine increased *Ins1* promoter activity while having modest positive effects on viability. Carbamazepine, isoconazole, allantoin, amitryptiline, nifedipine, metformin and ascorbic acid all increased *Ins1* promoter activity by these criteria, while having neutral effects on *β*-cell number (i.e. viability). Thus, there was excellent overall agreement between the two approaches. Electronic supplementary material, table S1 provides a full list of screen results.

As we have previously shown [23], a substantial portion of drugs in the Prestwick library of compounds is toxic to pancreatic *β*-cells (figure 3*c*). Interestingly, several factors, including cycloheximide, the established inhibitor of protein synthesis, increased *Ins1* promoter activity despite causing robust *β*-cell death. A few of the drugs (e.g. merbromin) are fluorescent, which interfered with the assay, and were ignored.

### 3.3 Follow-up validation of drugs that increase insulin expression

**Figure 4.**
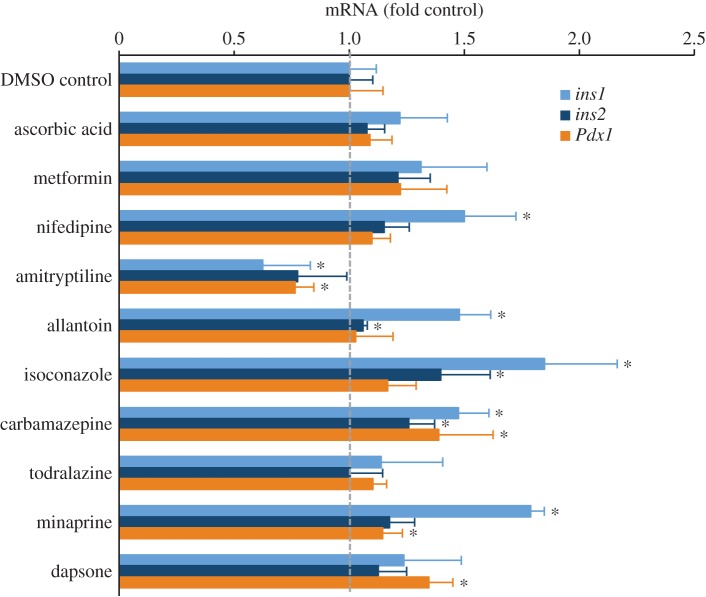
Validation of the effects of selected drugs on insulin gene expression using primary mouse islets. After 24 h of drug treatment, *Ins1*, *Ins2* and *Pdx1* gene expression were quantified with qRT-PCR relative to DMSO controls in primary mouse islets (*n*=8). *p<0.05.

In order to determine whether increases in *Ins1* promoter activity could be reflected in real changes in insulin mRNA, we conducted follow-up studies using validated qPCR primers [5,7,9,18]. For these experiments, we employed primary mouse islet cells, which represent a more physiologically relevant cell model than the MIN6 cell line. We examined the mRNA expression of both rodent insulin genes, *Ins1*and *Ins2*, as well as *Pdx1*, a transcription factor that supports insulin transcription, and is critical for adult *β*-cell function and survival [28,29]. Of the drugs chosen for follow-up, nine of 10 increased insulin mRNA expression in primary mouse islets (figure 4). Allantoin, isoconazole, minaprine and carbamazepine showed statistically significant increases in *Ins1* and/or *Ins2* mRNA expression above the DMSO control (figure 4). Carbamazepine increased *Ins1*, *Ins2* and *Pdx1* mRNA expression in these experiments (figure 4). Together, these results suggest that our nuclear-targeted *Ins1*/*Pdx1* dual-reporter system can identify novel regulators of insulin production.

**Figure 5.**
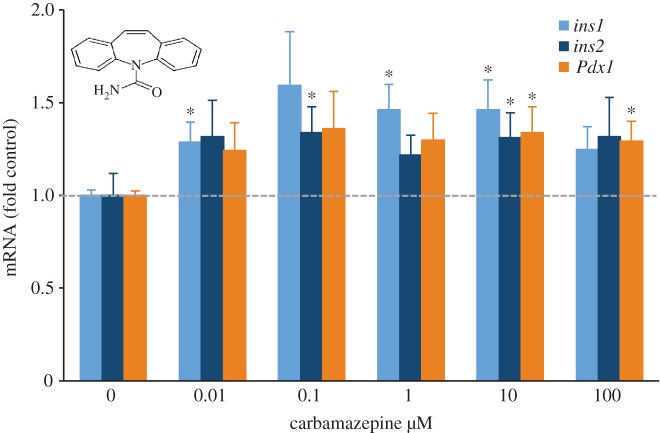
Dose–response profile of carbamazepine on insulin gene expression in primary mouse islets. After 24 h of carbamazepine treatment, *Ins1*, *Ins2* and *Pdx1* gene expression were quantified with qRT-PCR relative to DMSO controls in primary mouse islets (*n*=7). *p<0.05.

The observation that carbamazepine increased insulin expression was interesting, because we have recently implicated the same drug as protective in the context of a toxic cytokine cocktail [23]. Thus, we performed a more detailed dose–response analysis of the effects of carbamazepine on *Ins1*, *Ins2* and *Pdx1* mRNA expression in primary mouse islets. These experiments showed that carbamazepine increased *Ins1* mRNA in primary islets even at low doses (figure 5), orders of magnitude below the doses (100 μM) at which this drug has consistent, statistically significant effects on primary *β*-cell survival [23]. Thus, in our studies, carbamazepine may be a more potent regulator of insulin expression than of *β*-cell survival.

### 3.4 Carbamazepine directly inhibits the Nav1.7 sodium channel isoform

**Figure 6.**
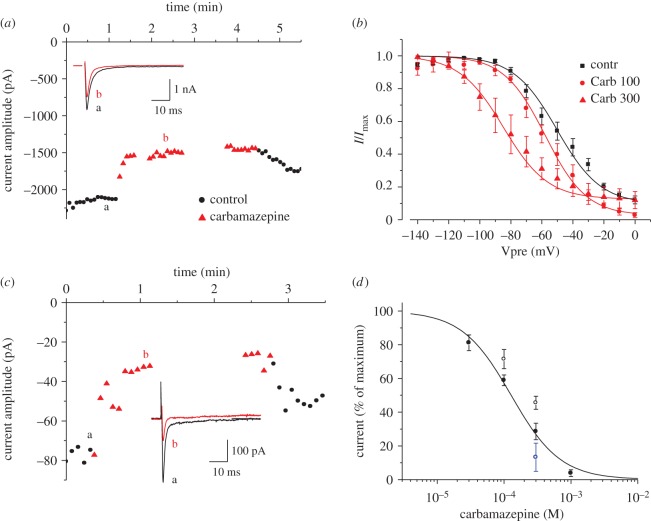
Carbamazepine inhibits Nav1.7 channels. (*a*,*c*) Nav1.7 current was measured from a holding potential of −80 mV in HEK 293 cells (*a*) or −120 mV in islet cells (*c*). Carbamazepine 100 (*a*) or 300 μM (*c*) inhibited the current reversibly. Insets show Na^+^ currents in the absence and presence of drug taken as indicated by the letters (a,b). (*b*) Steady-state inhibition of Nav1.7 in HEK 293 cells. The half-maximal inactivation was determined as *V*_0.5_ and was −50.2±3.8 for control and −57.8±3.8 and −84.7±7.5 for 100 and 300 μM carbamazepine, respectively. (*d*) Dose–response curves. In HEK 293 cells, cumulative inhibition was measured. The results from five to six cells per concentration were averaged and the curve calculated by a Hill equation (*solid line*). Similar results were obtained in isolated islet cells at HP −120 mV (*open circles*) or HP −80 mV (*open blue circle*).

We and others have recently characterized the expression and function of sodium channels in mouse pancreatic *β*-cells [23,24]. Gene expression analysis in purified *β*-cells revealed that the gene encoding Nav1.7 (*Scn9a*) was the most highly expressed of these channels [23,24]. Here, we determined that carbamazepine inhibits Nav1.7 Na^+^ currents (figure 6). Specifically, Nav1.7 channels expressed in HEK293 cells were dose-dependently and voltage-dependently inhibited by carbamazepine. The steady-state inactivation curve was shifted by more than 30 mV to hyperpolarized potentials in the presence of 300 μM carbamazepine (figure 6*a*,*b*). The half-maximal inactivation of Nav1.7 current in HEK 293 cells was IC_50_=130 μM. Similar observations under identical conditions were made in normal mouse islet cells. This effect was also voltage-dependent as shown by shifting the holding potential in islets from −120 to 80 mV (figure 6*c*,*d*).

### 3.5 Insulin production in sodium channel knockout mice

**Figure 7.**
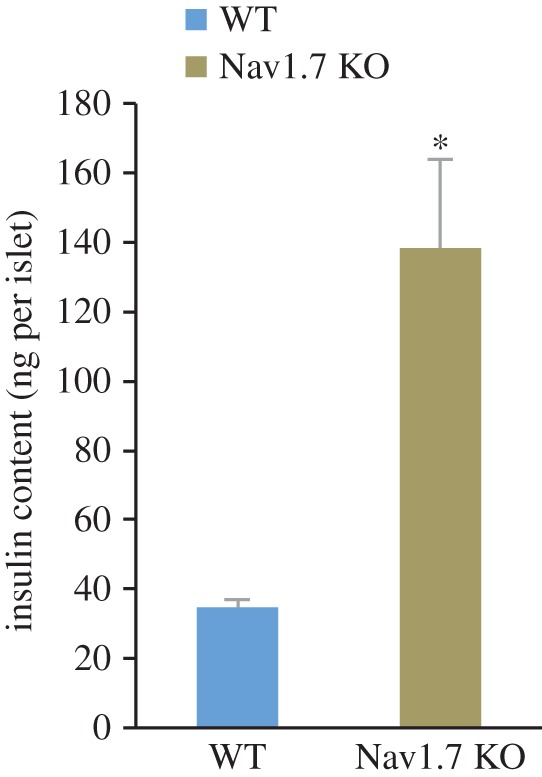
Cumulative effect of sodium channel loss-of-function on insulin production *in vivo*. Insulin content in islets from female, >1 year old, wild-type or Nav1.7 (*Scn9a*) knockout mice (*n*=5,6). *p<0.05.

We reasoned that if inhibition of *β*-cell sodium channels stimulates insulin gene expression *in vivo*, then mice lacking the most abundant sodium channel isoform would have accumulated increased levels of insulin protein within their islets. Indeed, and quite remarkably, isolated islets from Nav1.7 knockout mice had more than a threefold higher level of insulin content (figure 7). The striking elevation of insulin content contrasts with a relatively minor inhibition of glucose-stimulated insulin release from Nav1.7 knockout islets [24]. This observation strongly suggests that sodium channel activity specifically and negatively modulates insulin production *in vivo* over the long term.

## 4. Discussion

The goals of this study were to develop and validate an improved high-content screening-compatible *Ins1*/*Pdx1* ratiometric lentiviral reporter, and to use this fluorescent probe to identify drugs capable of modulating pancreatic *β*-cell differentiation and maturation. Indeed, our nuclear-targeted fluorescent reporters improved the ability to identify and count single cells, while retaining the fidelity of the original construct [5,7,9]. We expect that this improved dual-reporter lentivirus has the potential to be also useful for many studies on the fundamental biology of *β*-cell state and heterogeneity. Our high-content screening results demonstrate the use of this live-cell imaging tool for the identification of factors that increase insulin expression, and point to previously unexpected roles for ion channels in *β*-cell function.

Insulin production is a cardinal function of pancreatic *β*-cells and it is under tight control at transcriptional, translational and post-translational levels [30–32]. Insulin production is modulated by nutrients including glucose, and perhaps by insulin itself [29–31,33–36]. Here, we present evidence that several pharmacological compounds can affect insulin production. In many cases, it is the first evidence that a specific drug class that can affect *β*-cells. We observed statistically significant stimulation of *Ins1* mRNA expression with allantoin, isoconazole, minaprine and carbamazepine. Allantoin is also referred to as 5-ureidohydantoin or glyoxyldiureide, and is best known as a component of cosmetics. Isoconazole is an antifungal drug. Minaprine is a discontinued anti-depressant drug thought to work via reversible inhibition of monoamine oxidase A [37]. It is not likely that these three drugs have a path to clinical application for *in vivo* diabetes therapy, although the pathways they target might eventually be exploited *in vitro* to increase insulin yields for cell therapy applications.

Carbamazepine, on the other hand, may have therapeutic potential. Carbamazepine is a use-dependent inhibitor of sodium channels [23], and we have confirmed that it has this function in pancreatic *β*-cells. Mouse pancreatic *β*-cells have a relatively unique complement of sodium channel genes, with Nav1.7 (*Scn9a*) being the most prominently expressed mRNA [23,24]. Importantly, it appears that it may be possible to target these channels pharmacologically with only relatively modest effects on glucose-stimulated insulin secretion [23,24,38–42]. Indeed, this hypothesis was further supported by our observation that islets from lifelong Nav1.7 (*Scn9a*) knockout mice had increased insulin content, but relatively normal insulin secretion [24]. We have previously shown that carbamazepine, at concentrations much higher than those that modulate insulin production in this study, does not exhibit a robust, acute effect on insulin secretion in primary mouse islets using the gold-standard perifusion assay [23]. Thus, while chronically reduced insulin production can indirectly reduce insulin secretion [3,34], the converse is not true in the cases of carbamazepine and Nav1.7 knockout that increase insulin production, but do not increase insulin secretion [23,24]. Notwithstanding, a recent luciferase-based high-throughput screen identified Na^+^ channel modulators as potential regulators of insulin secretion in insulinoma cell lines [43], indicating that this area requires further study. It should be noted that a series of studies have shown that carbamazepine can have direct effects on K_ATP_ channels [44,45]. Given the importance of K_ATP_ channels in *β*-cell physiology [40], it is likely that this drug acts through multiple mechanisms to affect this cell type.

Collectively, our studies suggest that carbamazepine or derivatives, regardless of the exact molecular mechanism or mechanisms, may hold promise for increasing insulin production, promoting *β*-cell differentiation, and/or protecting *β*-cells from stress. Additional future work in this area, including *in vivo* experiments, is warranted.

## Supplementary Material

supplemental data
